# Genetic diversity of maize landraces from the South-West of France

**DOI:** 10.1371/journal.pone.0238334

**Published:** 2021-02-01

**Authors:** Yacine Diaw, Christine Tollon-Cordet, Alain Charcosset, Stéphane D. Nicolas, Delphine Madur, Joëlle Ronfort, Jacques David, Brigitte Gouesnard

**Affiliations:** 1 Institut Sénégalais de Recherches Agricoles, ISRA-CNRA de Bambey, Dakar, Sénégal; 2 AGAP, CIRAD, INRAE, Institut Agro, Univ Montpellier, Montpellier, France; 3 INRAE, CNRS, AgroParisTech, GQE—Le Moulon, Université Paris-Saclay, Gif-sur-Yvette, France; National Cheng Kung University, TAIWAN

## Abstract

From the 17th century until the arrival of hybrids in 1960s, maize landraces were cultivated in the South-West of France (SWF), a traditional region for maize cultivation. A set of landraces were collected in this area between the 1950s and 1980s and were then conserved *ex situ* in a germplam collection. Previous studies using molecular markers on approx. twenty landraces from this region suggested that they belonged to a Pyrenees-Galicia Flint genetic group and originated from hybridizations between Caribbean and Northern Flint germplasms introduced to Europe. In this study, we assessed the structure and genetic diversity of 194 SWF maize landraces to better elucidate their origin, using a 50K SNP array and a bulk DNA approach. We identified two weakly differentiated genetic groups, one in the Western part and the other in the Eastern part of the studied region. We highlighted the existence of a longitudinal gradient along the SWF area that was probably maintained through the interplay between genetic drifts and restricted gene flows. The contact zone between the two groups observed near the Garonne valley may be the result of these evolutionnary forces. We found in landraces from the East part of the region significant cases of admixture between landraces from the Northern Flint group and landraces from either the Caribbean, Andean or Italian groups. We then assumed that SWF landraces had a multiple origin with a predonderance of Northern Flint germplasm for the two SWF groups, notably for the East part.

## Introduction

Maize was domesticated from teosinte, *Z*. *mays* L. subsp. *parviglumis*, about 9000 years ago in Southern Mexico [1]. Thereafter, it spread from this domestication center over North and South America [1–3]. During these expansions, maize evolved in contrasted environments, leading to distinct genetic groups adapted to new climates such as cold temperatures and long days in the north and warmer temperatures in coastal Central America. After the discovery of America by Columbus in 1492, maize was introduced to Europe for the first time in 1493 from the Caribbeans to Spain [4–6]. In the first half of the 16th century, a second introduction of maize is documented in North-Eastern Europe from Northern Flint material originating from North America as suggested by an illustration of Leonard Fuchs, a German herbalist, in 1542 [4, 7]. Genetic and morphological differences between European maize landraces were found in numerous studies: studies based on morphological data separated European maize landraces principally according to their flowering time earliness and related morphological traits [8, 9], and maize landraces from North-Eastern Europe were shown to flower earlier than those from Southern Europe. Similarly, molecular studies confirmed a major differentiation between Northern and Southern Europe maize [4, 7–11]. Gauthier *et al*. [9] also detected genetic differentiation between South-Western (Northern Spain, Portugal and the Pyrenees) and South-Eastern (Greece and Italy) European landraces. These results suggested that the landraces cultivated in the North, South-West and South-East of Europe each resulted from introductions of different maize landraces belonging to different American regions.

Other molecular studies showed that maize landraces cultivated at intermediate latitudes in Europe originated from hybridizations between maize landraces from Caribbean, South American and North American landraces [4, 5, 7, 10–13]. These studies also revealed genetic similarities between American Northern Flint landraces and landraces from Northern Europe, and also between Caribbean and Southern Spain landraces, showing that the first maize landraces introduced from America still have direct representatives in Europe. By analysing both American and European landraces with SSR markers, Camus-Kulandaivelu *et al*. [10] were able to identify 7 genetic groups referred to as Mexican (MEX), Caribbean (CAR), Corn Belt Dent (CBD), Andean (AND), Northern Flint (NF), Italian Flint (ITA) and Pyrenees-Galicia Flint groups. Dubreuil *et al*. [7] found that maize landraces cultivated in Pyrenees and Galicia do not display close similarity with any of the American genetic group. Nevertheless, based on structure analyses, these landraces were shown to be intermediate between landraces from the South of Spain and from the North of Europe. Recently, Brandenburg *et al*. [12] analysed by resequencing first-cycle inbred lines directly derived from American and European landraces and showed that the European Flint group was close to the Northern Flint group. They also detected gene flow from ancestors belonging to Southern European landraces. Mir *et al*. [11] argued that landraces from Pyrenees displayed a hybrid origin between Northern U.S Flint and Northern South-American landraces, with a predominance of the latter. Thus, all these studies highlighted the importance of Northern Flint in the origin of the Pyrenees-Galicia group. The geographic origin of this NF material remains an open question, *i*.*e*. did it arrive directly from America or indirectly through NF landraces from the North of Europe. These two patterns of NF introductions were proposed by Tenaillon and Charcosset [13] on their map of maize introduction to Europe.

All the above information highlights the need for genetic markers analyses to better understand the evolution of maize landraces located in intermediate regions of Europe. Since maize is an outcrossing species, landraces are expected to display relatively high levels of genetic diversity. To assess this diversity, molecular studies must thus include several individuals. However, for large sets of landraces, molecular characterization based on several individuals per landrace is limited by laborious and costly experimental processes. To overcome this problem, several studies on maize landraces were performed on bulks of several plants per landrace, for RFLP analysis [4, 9, 14, 15] and SSR analyses [7, 10, 11, 16–19]. First, Dubreuil *et al*. [14] evaluated a method based on the RFLP analysis of balanced DNA bulks from several individuals. They found that allelic frequencies could be estimated with a high precision in bulks of 15 individuals. The major disadvantage of bulk DNA analysis was the loss of information on individual genotypes, preventing access to variation at individual level and thus to genetic parameters such as Wright’s fixation indices (*F*_IS_ and *F*_IT_). Nevertheless, bulking is highly efficient to estimate most parameters of interest regarding population genetic structure and to make inference about the evolutionary history of populations. Recently, this bulk approach was investigated on SNP markers in maize [20], leading to the development of a new method for allelic frequency estimations [21].

In this report, we focus on the South-West of France (SWF), one of the main traditional maize cultivation areas in Europe. Maize cultivation in SWF was first reported in 1626 in the ‘Béarn’ region, and in 1628 and 1637 respectively in the towns of ‘Bayonne’ and ‘Castelnaudary’ [22]. Based on historical records, maize spread from its introduction in the South-West of France at the end of the 16th century and was already largely cultivated in this area (from the Pyrenees to the Garonne Valley) at the end of the 17th century [22]. American hybrids were introduced from 1948 onwards in France and maize landraces in SWF were progressively replaced by commercial American x European hybrids. The origin and genetic diversity of these landraces before the introduction of commercial hybrids are poorly known. The aims of this paper are (1) to assess the structure and genetic diversity of traditional maize landraces cultivated in the South-West of France until the 1970s, and (2) to determine genetic relationships between SWF landraces andAmerican and European maize landraces. To do so, we used the 50 K SNPs array [20, 21, 23] to analyse the diversity of 342 maize landraces, including 194 landraces collected in the South-West of France and conserved by INRA since the 1960s; as well as 148 European and American maize landraces already analysed by Camus-Kulandaivelu *et al*. [10] and Mir *et al*. [11] using SSR markers and by Arca *et al*. [23] using SNP analysis.

## Materials and methods

### Plant material

We studied 194 landraces that had been collected between 1949 and 1987 [24, 25] in the South-West of France (SWF, S1 Table) in the two French administrative regions “Nouvelle Aquitaine” (in 5 districts) and “Occitanie Pyrénées-Mediterranée” (in 9 districts). Since their collection, these landraces have been preserved at the French Maize Biological Resource Center (CRB, Mauguio, France, https://urgi.versailles.inra.fr/siregal/siregal/grc.do) and seed lots regenerated through four successive generations of multiplication using between 100 and 200 full-sib ears for each landrace. Passport data including information about the area of collection (continent, country and district) and geographical coordinates (latitude and longitude) are available for each landrace although geographic coordinates are lacking for 36 of these 194 landraces (S1 Table).

To address questions about the origin of these landraces and to compare landraces from SWF with original American material, we used a worldwide reference panel consisting of 64 European and 73 American maize landraces (S2 Table) previously analysed using SSR markers by Camus-Kulandaivelu *et al*. [10] and using SNP analysis by Arca *et al*. [23]. These authors identified 7 genetic groups for these maize landraces that they termed the Andean (AND), Caribbean (CAR), Mexican (MEX), Northern Flint (NF), Corn Belt Dent (CBD), Italian (ITA) and the Pyrenees-Galicia (PG) groups. The NF, ITA, CBD and PG groups included respectively 2, 1, 2 and 17 landraces from the French Pyrenees. These 22 landraces originating from the region focused in this paper, we chose to remove them from their reference groups and put them in the landraces of the SWF region to assess *a posteriori* their genetic proximity with the other groups. Thus, the size of each reference groups was finaly as follows: 12 for AND landraces, 25 for CAR, 22 for MEX, 37 for NF, 15 for CBD, 17 for ITA and 9 for Pyrenees_Galicia _2 (PG_2) groups (S2 Table). The PG_2 reference group comprised 9 landraces from the PG genetic group identified by Camus-Kulandaivelu *et al*. [10] that originated from outside SWF. To complement this reference set, we added 2 landraces from Portugal and 9 landraces from South America previously analysed by Mir *et al*. [11].

### DNA extraction and genotyping with 50K SNP array

We assessed nucleotide diversity in the 194 maize landraces collected in South-West France using the Maize 50K SNP array developed by Ganal *et al*. [20] and DNA bulks representing each landrace. To this aim, DNA was extracted from leaf disks collected on 15 individuals per landrace. To assess population diversity, we selected the 30,068 Panzea markers (PZE-prefix SNPs) proven suitable for diversity analyses [20] and we predicted SNPs allele frequencies using the method described in Arca *et al*. [21] briefly presented below. The approach consists in a two-steps analysis of the relative fluorescence ratio for each allele. The first step consists in determining, for each SNP, whether a landrace is fixed for the A (or for the B) allele, by comparison with the distribution of ratio of A *vs*. B fluorescence within a set of 327 inbred lines. If the SNP is declared polymorphic within the landrace, the second step consists in estimating the allelic frequencies at this locus using a calibrated average curve established from1000 SNPs on polymorphic DNA pools controlled for their composition. Arca *et al*. [21] observed that the mean absolute error (MAE) for allelic frequencies estimation at both steps was on average 7%. For the 82 American and the 66 European maize landraces used as a worldwide reference panel, we used Arca *et al*. [21]'s SNPs database obtained using the same SNP array and following the same procedure. Combining these different datasets, we finally obtained an allele frequency database for 23,412 SNP in which diversity was assessed on 342 landraces.

### Diversity and genetic structure analysis of SWF landraces

For each landrace, we estimated the allelic richness (*A*_r_) and gene diversity (*H*_e_, [26]) using a *ad hoc* script in the R language v3.0.3 [27].

To assess the population genetic structure underlying our panel of SWF maize landraces, we used the software ADMIXTURE v1.07 [28]. This method assumes the existence of a predetermined number (K) of clusters and estimates the fraction of ancestry of each accession in each of the K clusters (Q). It also infers the SNP allele frequencies of the ancestral landraces (P). The software requires individual genotypic data, while our SNP analysis on DNA bulks provides allelic frequencies only. To obtain individual genotypic data, we thus simulated 5 haploid genotypes per landrace from allele frequencies under the hypothesis of Hardy-Weinberg equilibrium and no linkage disequilibrium [10, 21, 23]. To limit linkage disequilibrium between SNPs, we divided the maize genetic map into 2500 non-overlapping windows and randomly selected a single SNP in each windows [21, 23]. We explored K values ranging from K = 1 to K = 13. The likely number of genetic groups was estimated using the *DeltaK* parameter following the method proposed by Evanno *et al*. [29]. Each landrace was assigned to the cluster where its fraction of ancestry was highest.

### Relationship between landrace genetic structure, geographic and climatic variables

To describe the genetic structure of SWF landraces, we first looked for links between the genetic structure and the geographic localization of the landraces. To this aim, we calculated pairwise genetic distances over the 194 maize landraces using the modified Rogers’s distance [30, 31]. On the corresponding genetic distance matrix, we performed a principal coordinate analysis (PCoA) using R ade4 package and estimated correlations between landrace coordinates on the first axis of the PCoA and either (1) the longitude or (2) the latitude of their site of collection. This analysis was based only on those 158 SWF landraces with available geographic coordinates. We also looked for patterns of isolation by distance. To this aim, we performed a regression analysis between the “genetic divergence matrix” which we estimated using the linearized *F*_ST_ /(1- *F*_ST_) values following Rousset [32], and the pairwise geographic distance matrix calculated using latitude and longitude coordinates of the site of colection of each landraces with spherical law of cosines formula [33]. Pairwise *F*_ST_ values were calculated over SWF landraces using the Gst estimate proposed by Nei [34]. The statistical significance of the correlation between these two matrices was evaluated using a Mantel test [35], R vegan package.

We also explored relationships between the genetic structure and climatic variables. To this aim, we created a climatic data base containing values of monthly precipitation and monthly mean temperatures of prospection sites for the 194 landraces from the SWF. We first retrieved data for monthly precipitation and monthly minimum and maximum average temperatures from the WORLDCLIM database [36]. Then, we estimated the monthly mean temperatures as mean of the sum of monthly minimum and maximum average temperatures. Considering that maize was cultivated in Europe from May to October, we retained the mean monthly temperature and mean monthly precipitation for only these 6 months. These lead us to obtain a climatic database of 12 variables of maize growing season. When we performed a principal component analysis on these 12 variables with R ade4 package, we observed that the first axis of this PCA explained 82.5% of the climatic variability observed for temperatures and precipitations covered by the locations of our set of 158 SWF landraces. The climatic matrix distance between our 158 landraces was computed as the Euclidian distance on this first PCA axis (Ecodist R package, Goslee and Urban [37]). Thereafter, we used the MRM function of the Ecodist R package [37] to perform a multiple regression of the genetic distance matrix on the geographical and the climatic matrixes. We also compared, through an ANOVA analysis, the climatic characteristics of the different genetic groups identified using ADMIXTURE.

### Comparative analyses between SWF landraces and worldwide maize genetic groups

#### Genetic diversity

To compare allelic richness and gene diversity between well-known maize genetic groups and our set of SWF landraces, we used results obtained using ADMIXTURE on our panel of 194 maize landraces for K = 2 and considered the seven genetic groups identified by Camus-Kulandaivelu *et al*. [10]. Allelic richness and genetic diversity (*H*_e_) were estimated in each of these groups and, to compare allelic richness (*A*_r_) among groups of different sizes, we used the rarefaction method proposed by Petit *et al*. [38] with *n* = 1000 resampling. To estimate the expected heterozygosity (*H*_e_) for each group, we calculated the averaged values of allelic frequencies at the 23K SNP over the landraces owing to each group. Pairwise comparisons of *A*_r_ and *He* values between groups were carried out using Wilcoxon signed-rank tests across all the 23 K SNPs. Statistical analyses were computed using a R Core Team [27] script (S. Nicolas, personal communication).

#### Looking for footprints of hybridization between main maize genetic groups in the origin of SWF landraces

To determine whether SWF landraces originated from hybridizations between ancestral landrace groups, we used the 3-populations test [39] implemented in the TreeMix software version 1.12 [40] This test compares a focus population (or group) X to two reference populations (or groups) Y and W, and calculates an *f3 statistic*, f3(X; Y, W) defined as the product of the difference in allele frequencies between populations X and Y, and the difference in allele frequencies between populations X and W. If the focal population X can be considered as resulting from an admixture or hybridization event between populations Y and W, the value of the *f3-stat* can be negative. A *Z-score* value <-2 indicates a significant mixture [39].

In our analysis, we tested two scenarios for the origin of SWF landraces. In the first one, we made the hypothesis that SWF landraces originated from hybridizations between maize landraces from Caribbean (CAR) and Northern Flint (NF) genetic groups as proposed in previous articles. To this aim, we applied 3 population s tests on triplets composed of a focal SWF group previously identified using ADMIXTURE analyses and the two genetic groups representing CAR and NF. In order to test the distinct involvement of NF materials from North America and NF from Northern Europe in the origin of SWF landraces, we subdivided NF landraces into American Northern Flint (NFA) and European Northern Flint (NFE) groups. Then, we analysed two hypotheses: SWF landraces originate from (1) an hybridization between Caribbean landraces and Northern Flint landraces previously introduced in Europe (NFE), or (2) an hybridization between Caribbean landraces and American Northern Flint landraces (NFA) potentially independently introduced in the South-West of France.

In the second scenario, we assumed that SWF landraces originated from hybridizations but without any *a priori* about the parental groups. For example, to determine whether SWF landraces originated from hybridization between landraces belonging from NF and MEX groups, we performed TreeMix 3-population tests using each of the genetic groups identified in the SWF as the focus group and NF and MEX groups as the parental groups. All these groups were defined on the basis of structure analysis using the arbitrary cut off of upper than 0.7.

To vizualize genetic relationships between SWF landraces and worldwide reference genetic groups, we also performed a Neighbor joining tree with ape R package and a principal component analysis (PCA) with ade4 R package. For both analyses, we used the modified Rogers’ genetic distance matrice [30] estimated on SNP data of 342 maize landraces. To perform the PCA analysis, we only used the 72 American landraces representing the 4 main historical maize genetic groups (*i*.*e*. MEX, AND, CAR and NF groups). The European maize landraces (including SWF landraces) were added as supplementary data. Due to their recent origins, the landraces of CBD group [41] were also added as supplementary data. We used the modified Rogers’ distance between each SWF landrace and each American landrace in order to identify the closest American maize genetic group.

## Results

Among the 23412 SNPs analysed, 378 exhibited missing data for at least one landrace and discarded, leaving 23034 SNPs. Among them, 15 were monomorphic in all the landraces considered in this study (*n* = 342). At landrace level, the proportion of polymorphic loci varied from 13.5% for IPK60, a German Northern Flint landrace, to 92.8% for EPZMV23, a landrace from Spain. On average, the proportion of polymorphic loci within landrace was 68.7%.

### Genetic diversity and population structure

**Fig 1:**
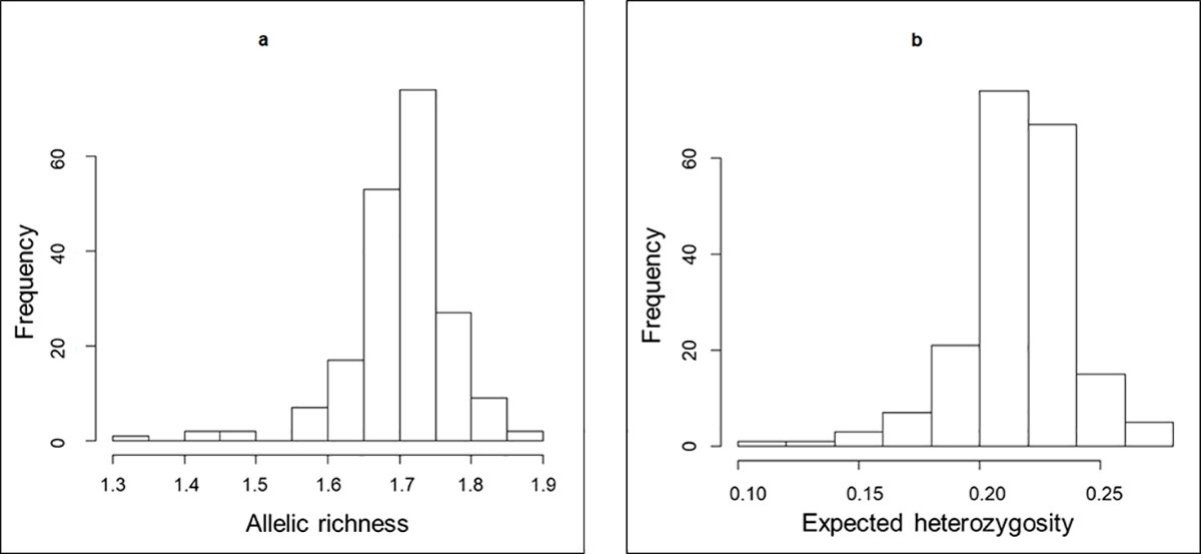
Genetic diversity analysis of SWF landraces. Histogram of (a) allelic richness and (b) expected heterozygosity values estimated for each of the 194 landraces.

The proportion of polymorphic SNPs in landraces from the South-West of France varied between 34% and 86%, with an average value of 70%. Averaged over all SNPs, the mean allelic richness (*A*r) per landrace was 1.70, and varied from 1.33 to 1.86. Mean gene diversity (*H*_e_) was 0.22 per landrace (min = 0.12; max = 0.27, Fig 1, S1 Table).

**Fig 2:**
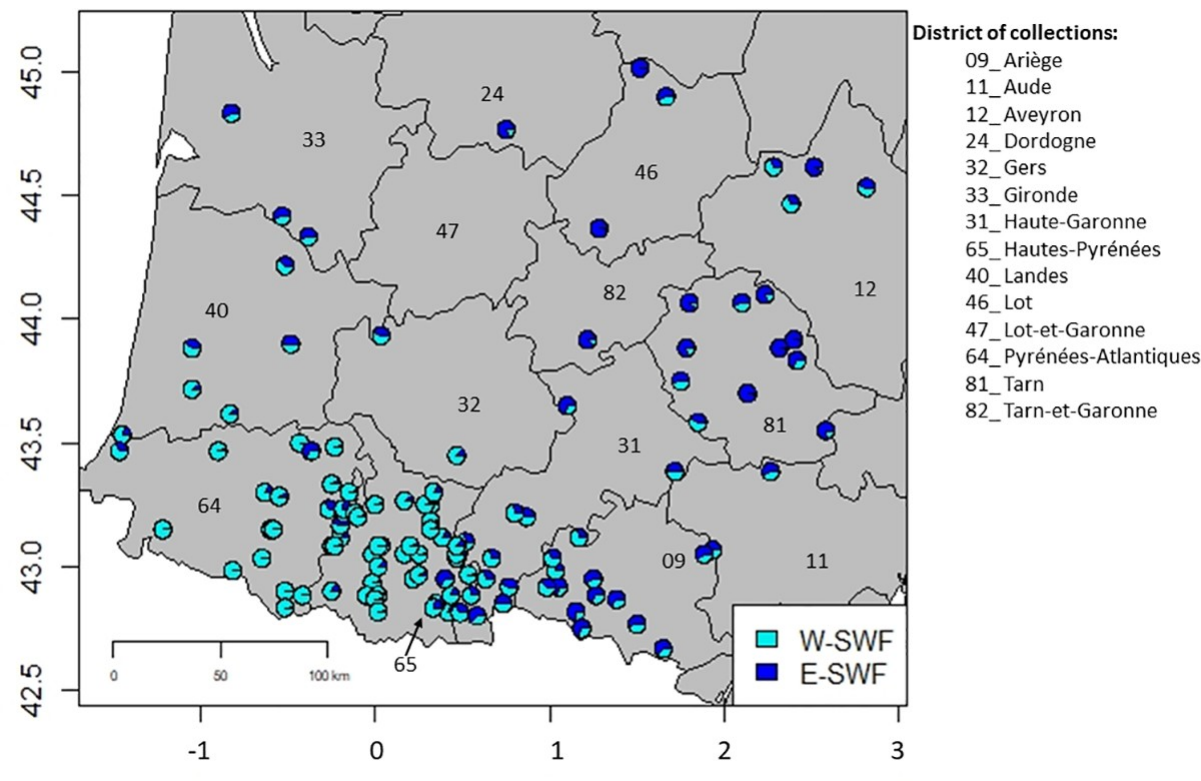
Geographical representation of the 158 maize landraces from the South-West of France (36 landraces having no geographical coordinates were not plotted). The districts of collection sites are represented with administrative numbers. Landraces for which pie-diagrams had both blue and cyan colors exhibited mixed genetic origins between E-SWF and W-SWF groups. The map was created using R software (libraries maps, plotrix, mapproj, devEMF).

Using the ADMIXTURE software on the whole set of SWF landraces, we observed that the maximal deltaK value occurred at K = 2 (S1 Fig). The next two large peaks of deltaK value were observed at K = 3 and K = 5 (S1 Fig). As shown in Fig 2, we identified a stratification in two major groups at K = 2 distinguishing landraces located in the Eastern part of the South-West of France from those located in the Western part. The first group, which was named ‘East South-West France’ (E-SWF), included 65 landraces. Most of these landraces were collected in Eastern districts such as Ariège, Tarn and Haute-Garonne, but ten of them were collected in Western districts (*i*.*e*. districts such as Pyrénées Atlantiques, Hautes-Pyrénées, Landes and Gironde). The second group, referred to hereafter as ‘West South-West France’ (W-SWF), included 126 landraces mostly originating from the Hautes-Pyrénées, Pyrénées-Atlantiques and Landes districts. Twenty-three landraces from this group were nevertheless collected in the Eastern part of the area. Interestingly, most of the landraces collected in the Haute-Garonne and Ariège districts exhibited patterns of admixture between W-SWF and E-SWF groups (Fig 2). The contact zone between E-SWF and W-SWF groups is located between these two districts, near Garonne valley (Fig 2). At K = 3, we observed a third group mostly composed of the 10 landraces that were previously assigned to E-SWF group at K = 2 but located in the Western part of the region (S2 Fig). At K = 5, we observed two groups, E-SWF and W-SWF, a new group shaped by landraces from Gironde and Lande districts (12 and 4 respectively), and two other groups mostly deriving from the third group identified at K = 3.

**Fig 3:**
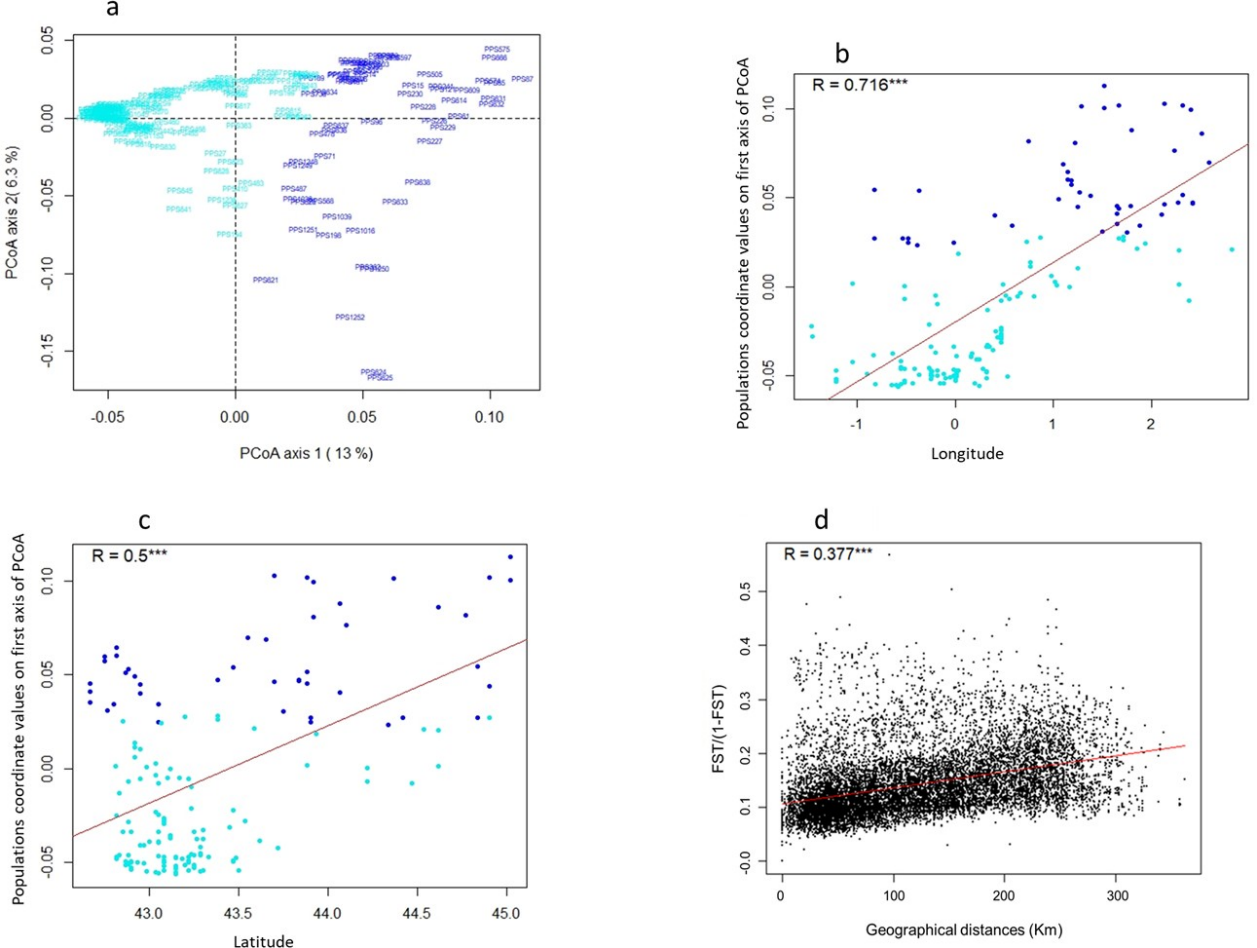
Spatial genetic structuration analysis of SWF landraces. (a) Principal coordinate analysis on SNP data using Rogers ‘genetic distance matrix estimated among the 194 landraces. (b) Longitudinal and (c) latitudinal gradient analyses on SNP data of 158 landraces from South-West France using coordinates values on the first axis of PCoA analysis. SWF landraces were colored according to their assignation to E-SWF and W-SWF groups identified with admixture analysis at K = 2. (d) Isolation by distance (IBD) analysis of 158 landraces from South-West France using *F*_ST_ / (1- *F*_ST_) matrix for genetic differentiation estimated following Rousset [32] and geographical distance matrix estimated for each pair of landraces. R = coefficient of correlation. *** = p-value<0.001.

PCoA on allele frequencies revealed the same population structure pattern. The first axis (representing 13% of the total inertia) separated landraces located in the Western part (W-SWF cyan colour in Fig 3A) from those located in the Eastern part (E-SWF dark blue colour in Fig 3A). The second PCoA axis of Fig 3A (representing 6.3% of total inertia) highlighted variation among E-SWF landraces. Six landraces, assigned to the third group identified with ADMIXTURE analysis on SNP data of 194 SWF landraces at K = 3 (S2 Fig), were indeed strongly differentiated from the majority of E-SWF landraces on the PCoA plan 1–2. Fig 3B and 3C highlighted the existence of a longitudinal and a latitudinal gradient (respectively) along the SWF. Significant correlation was found between genetic differenciation based on F_ST_ and geographical distance (Fig 3D).

We analysed the genetic diversity of both groups. The allelic richness of W-SWF was significantly higher than that of E-SWF: *A*_r_ = 1.718 and *A*_r_ = 1.679 respectively (Wilcoxon’s signed-rank test, *p-value* < 10^−15^; Table 1). On the other hand, the E-SWF group exhibited a higher level of gene diversity compared to the W-SWF group: *H*_e_ = 0.290 and *H*_e_ = 0.273 respectively (*p-value* < 10^−15^ using Wilcoxon’s signed-rank test). The fact that *A*_*r*_ is lower and *H*_*e*_ is higher in E-SWF group compared to W-SWF group indicates that allelic frequencies are more balanced in E-SWF group than in W-SWF group. The W-SWF/E-SWF differentiation explained a low but significant proportion of the overall genetic diversity: *F*_ST_ = 0.026 (*p-value* < 10^−15^ based on permutation test over landraces). The mean *F*_ST_ between pairs of landraces was 0.104 and 0.168 for the W-SWF and E-SWF groups, respectively. This shows that the E-SWF landraces are, on average, more differentiated from each other than W-SWF landraces.

**Table 1:** Allelic richness (*A*_r_) and gene diversity (expected heterozygosity, *H*_e_) for the two SWF groups identified with admixture at K = 2 and the 7 genetic groups previously identified in Camus-Kulandaivelu *et al*. [10].

Group.Name	Population Size	Allelic richness (*A*_r_)	Expected heterozygosity (*H*_e_)
Andean	12	1.75	0.31
Caribbean	25	1.72	0.32
Corn Belt Dent	15	1.75	0.33
Pyrenees_Galicia_2	9	1.76	0.30
Italian Flint	17	1.66	0.29
Mexican	22	1.71	0.33
Northern Flint	37	1.52	0.27
W-SWF	129	1.72	0.27
E-SWF	65	1.68	0.29

*A*_r_ values were calculated using a rarefaction method as in [38], with 1000 resampling of 9 landraces without replacement in each group, except for the Andes group for which we obtained only 220 possible resampling of 9 landraces. We used a Wilcoxon signed-rank test to analyze statistical differences between peers of *H*_e_ (or pair of *A*_r_) values estimated for the 9 groups. All comparisons for pair of *H*_e_ (and pair of *A*_r_) were significant (*p-value*< 0.009); except for comparison between He of ITA and SWEF groups (*p-value* = 0.45) and comparison between *A*_r_ of Andean and Corn Belt Dent groups. E-SWF = East South-West France; W-SWF = West South-West France.

### Relationships between geographic distribution, climate variables and population structure

To determine the main factors underlying the genetic structure observed in SWF land races, we searched for a relationship between genetic variation and either the geographical origin of the landraces or the climatic characteristics of their site of origin. First, we looked for associations between (i) landrace coordinates on the first axis of the PCoA (Fig 3A) performed on SNPs and (ii) either the latitude or longitude coordinates of their prospection sites. As shown on Fig 3B and 3C, linear regression analyses evidenced significant correlations with both longitudinal (*r* = 0.72; *p-value* < 10^−15^) and latitudinal coordinates (*r* = 0.5; *p-value* = 10^−10^), highlighting the existence of both longitudinal and latitudinal genetic gradients. We also identified a significant correlation between the pairwise *F*_ST_ / (1- *F*_ST_) ratio and pairwise geographic distances (*r* = 0.38, *p-value* < 10^−15^, Fig 3D), suggesting isolation by distance.

Examining climate characteristics from May to October in the collection sites of our SWF landraces, we observed that landraces belonging to the E-SWF group originated from sites associated with higher temperatures and weaker precipitations than for W-SWF landraces (S3 Table). Differences in monthly temperatures and precipitations between E-SWF and W-SWF landraces were low, but significant variations were observed for temperature (from June to August) and precipitations (during September and October; see S3 Table, in bold). To explore how much genetic differentiation between SWF landraces could be explained by geographic distance instead of climatic distance, we performed multiple regression analyses of genetic distance on geographic and climatic distance matrices that showed a significant relationship only for the geographic matrix (*p-value* = 0.01 for geographical values VS, *p-value* = 0.67 for climatic values). This suggests that the demographic history (instead of selection) of these populations is the main factor driving population differentiation.

### Genetic diversity of SWF landraces compared to the main maize genetic groups

As shown in Table 1, the two genetic groups identified in the South-West of France using ADMIXTURE exhibited larger allelic richness (*A*_r_) and gene diversity (*H*_e_) than the Northern Flint group, but lower allelic richness and gene diversity than the Corn Belt Dent (CBD), Andean (AND) and Caribbean (CAR) groups.

#### Do SWF landraces originate from hybridization between known maize genetic groups?

We used a treeMix 3-population test to determine whether E-SWF and W-SWF groups result from hybridization events beween historical maize genetic groups. Two different hybridization scenarios were considered: (1) SWF landraces originated from hybridizations between landraces from Caribbean (CAR) and Northern Flint (NF) groups, or (2) SWF landraces originated from hybridizations between any of the historical genetic groups other than CAR x NF. Negative *f3-stat* values, suggestive of a hybridization event between two reference groups as ancestors, were detected for scenarios 1 and 2 for the E-SWF group (Table 2) but not for the W-SWF group (S4 Table). Z-scores of 3 populations tests showed that the most significant scenarios were mixed events between (i) landraces from NFA and (ii) landraces from either CAR or ITA groups (bold and italic line in Table 2). No negative f3-stat values were observed for W-SWF group.

**Table 2:** Negative values of f3-stat and Z-score on TreeMix 3-population test with E-SWF group as focus groups.

Focus group	Reference group 1	Reference group 2	f3-stat	Sd	Z-score
E-SWF	AND	NFA	-0.00314147	0.000766376	-4.09912
***E-SWF***	***CAR***	***NFA***	***-0*.*00897185***	***0*.*0008593***	***-10*.*4409***
E-SWF	CAR	NFE	-0.00739079	0.00082723	-8.93438
***E-SWF***	***ITA***	***NFA***	***-0*.*00874928***	***0*.*000730175***	***-11*.*9825***

AND = Andean, CAR = Caribbean, ITA = Italian, NFA = Northern Flint landraces from America, NFE = Northern Flint landraces from European. No negative f3-stat values were observed for the W-SWF group.

We did not observe signals for hybridization events involving CBD or MEX groups as origins of E-SWF and W-SWF groups, suggesting that these were not influenced by introductions from MEX landraces, nor by hybrids originating from CBD landraces after their introduction to Europe.

#### Genetic relatedness between SWF landraces and maize from Europe and America

**Fig 4:**
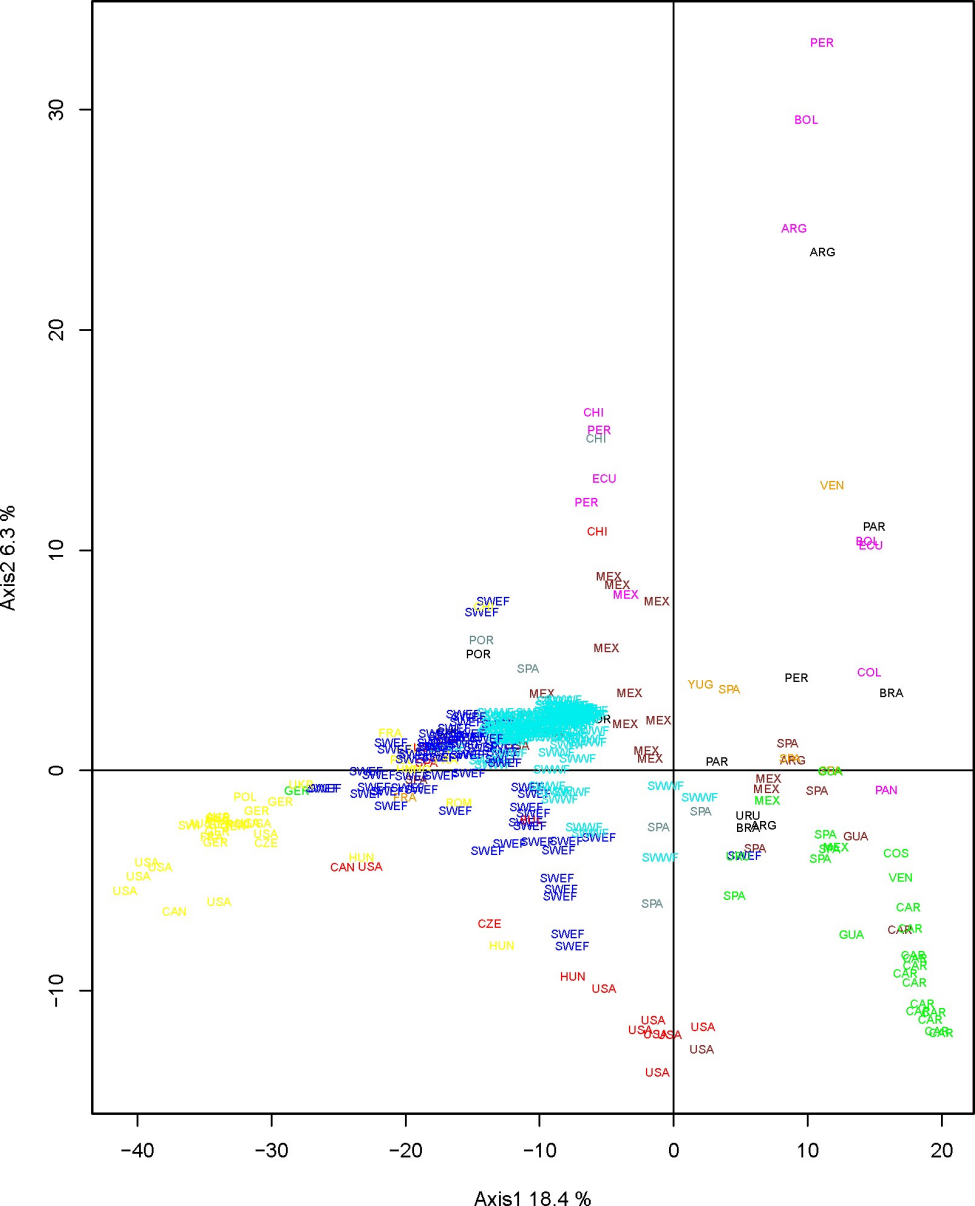
PCA analysis on SNP data of 72 American maize landraces with 260 maize landraces from Europe and those of CBD as supplementary data. Labels indicate landrace country of origins (see S2 Table). South-West France landraces were colored according to their admixture result at K = 2 allowing to distinguish East (in blue) and West South-West France (in cyan) genetic groups. The remaining American and European landraces were colored according to their genetic groups previously identified by Camus-Kulandaivelu et al. [10]: Corn Belt Dent in red, Caribbean in green, Northern Flint in yellow, Mexican in brown, Italian Flint in orange, Andean in magenta, Pyrenees_Galicia_2 in grey. The 9 landraces from South America and 2 landraces from Portugal studied by Mir et al (2017) were colored in black.

Results of the PCA analysis performed on SNPs for the whole set of 342 maize landraces are presented in Fig 4 and S2 Table. Fig 4 shows the position of the different groups on the first and second axes of the PCA. The first axis (19.7% inertia) mainly separated Northern Flint landraces (NFA, NFE) from Tropical landraces (AND, CAR, MEX) while the second axis (6.4% inertia) mainly separated South American from Caribbean landraces. As expected, most European NF landraces were close to American NF landraces, and landraces from Southern Spain were close to those from the Caribbean Islands. The two SWF groups occupied a central position on the first PCA plan (Fig 4) but were somewhat separated on the first two axes. Landraces from the E-SWF group were more dispersed than those from W-SWF. On the first axis, E-SWF landraces appeared closer to the NF group than did W-SWF landraces. On the contrary, W-SWF landraces were closer to tropical landraces (AND, CAR and MEX) than to NF landraces (Fig 4). As to relationships between landraces from Europe, we found that landraces from SWF were closer to Pyrenees_Galicia_2 (PG_2) and European Northern Flint landraces (NFE) than to Italian Flint landraces.

**Fig 5:**
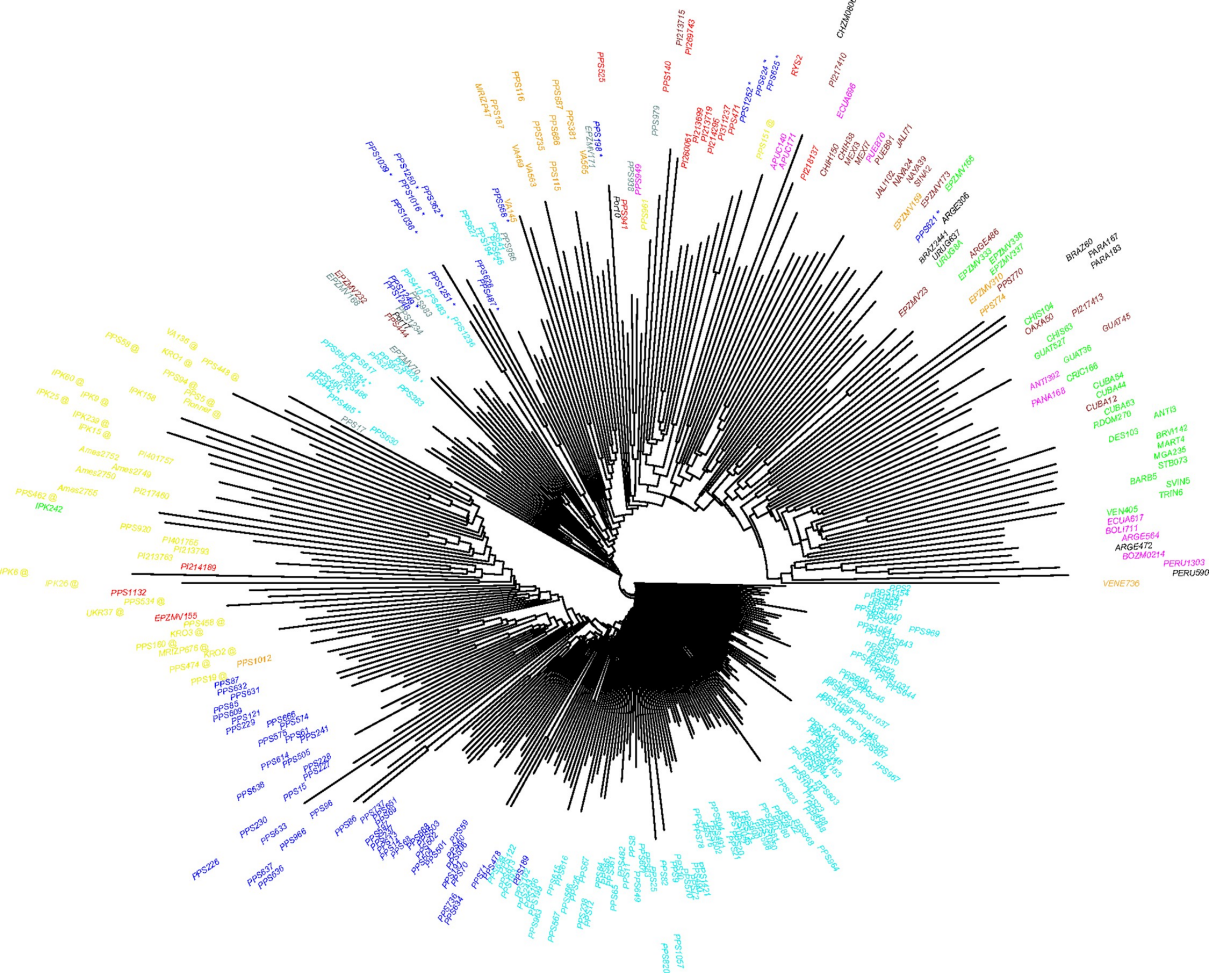
Neighbor joining tree for the 342 populations obtained from Rogers distance matrix. South-West France landraces were colored according to their admixture result at K = 2, allowing to distinguish East (in blue) and West South-West France (in cyan) genetic groups. The remaining American and European landraces were colored according to their genetic groups previously identified by Camus-Kulandaivelu et al. [10]: Corn Belt Dent in red, Caribbean in green, Northern Flint in yellow, Mexican in brown, Italian Flint in orange, Andean in magenta, Pyrenees_Galicia_2 in grey. The 9 landraces from South America and 2 landraces from Portugal studied by Mir et al (2017) were colored in black. SWF landraces assigned to the 3 other groups identified with our ADMIXTURE analysis on SNP data of 194 SWF landraces at K = 5 were represented with star symbols in blue and cyan. The European Northern Flint landraces (NFE) were represented by an @ symbol in yellow.

To further analyse the relationship between landraces from the SWF groups and reference groups, we performed a neighbor-joining tree based on Roger’s distance matrice on SNPs data of 342 landraces (Fig 5). We observed that landraces are distributed in two large groups distinguishing NF landraces from those from tropical regions of America (AND, MEX and CAR groups). The majority of landraces from SWF assigned to E-SWF and W-SWF groups were close to the NF group, suggesting a major influence of NF landraces in the evolution of SWF landraces. Landraces of E-SWF group were closer to NF group than landraces of W-SWF group. The remaining SWF landraces (36 landraces in total, blue and cyan star symbols in Fig 5) were close to landraces from the ITA, PG_2 and CBD groups, suggesting possible recent gene flow between these maize landraces from SWF and those from ITA and CBD groups. The ITA, PG_2 and CBD groups were themselves closer to landraces from Tropical region of America (AND, MEX and CAR groups). We also observed that these 36 landraces from the SWF were assigned to different genetic groups in our ADMIXTURE analysis with SNP data from 194 SWF landraces at K = 5, distinguishing them from E-SWF and W-SWF groups (S2 Fig).

To identify genetic distances between landraces from SWF and those from America, we used a heatmap representation with R gg2plot function to visualize the result of genetic distance estimation between SWF landraces and the 82 American landraces (S3 Fig). The heatmap representation of genetic distances showed that the 194 landraces from SWF were closer to 4 Chilean (PPS949 from AND, PPS941 from CBD, PPS961 from NF and PPS938 from MEX groups), 2 CBD (PI214189 and PI280061) and 2 NF (PI213793 and PI401755) landraces from North of America than landraces from others American countries. These results suggested an introduction of Northern Flint and CBD landraces from Northern America into the SWF genepool, as well as exchanges of maize landraces between Chile and SWF. Comparing the genetic distances of the two SWF groups with the American accessions, we did not evidence any clear difference between the genetic origins of these two groups, except that E-SWF landraces appeared to be close to landraces from the NF group and that W-SWF landraces were closer to Tropical landraces (S3 Fig).

## Discussion

### Validity and limitations of bulk DNA analysis with the 50 K SNP array

The usefulness of the bulk DNA approach in genetic diversity investigations has been proven in numerous studies [8, 14, 17, 18, 21, 42]. The major constraint observed in the use of this pooled DNA analysis is the loss of information about genetic variation at the individual level [8, 15]. As a result, genetic parameters such as individual’s heterozygosity and Wright’s *F*_*IS*_ fixation indices [31, 34] cannot be estimated directly. Bulk analysis also limits the use of classic genetic tools that require individual genotype information such as the ADMIXTURE [28], GENEPOP [43], HZAR [44] and STRUCTURE [45] softwares. As a result, we chose to estimate genetic parameters and to use software tools that do not require individual genotype information to characterize and analyse these landraces, except for the ADMIXTURE software for which we simulated genetic haplotypes [28].

Despite these constraints, the genetic proximities we revealed between American and European landraces using the 50K SNPs on DNA bulks were in accordance with what is known about maize diffusion in America and the main paths of maize introductions to Europe. Indeed, our PCA and Neighbor-joining analyses allowed distinguishing between the main historical maize genetic groups previously identified by Camus-Kulandaivelu *et al*. [10] using SSR markers. For instance, the genetic similarity between landraces from the South of Spain and Caribbean landraces corroborates the introduction of maize by Columbus from the Caribbean Islands to Spain [4–6]. We also detected strong genetic proximity between Northern Flint and Northern Europe landraces, in accordance with the introduction of maize landraces from Northern America to Northern Europe as suggested by an illustration of Leonard Fuchs, a German herbalist, in 1542 [4, 7].

### Landraces of South West of France are differenciated along a longitudinal gradient

Using a genetic database of 23412 SNP markers to analyse the genetic diversity of maize landraces that were collected in the South-West of France circa 50 years ago, we were able to identify two main groups of landraces, located respectively in the Eastern and in the Western side of the South-West of France. This differenciation was consistently found in the different analyses (ADMIXTURE, PCoA, PCA and neighbor-joining tree) even if the genetic differentiation between them was relatively low (*F*_ST_ = 0.03). These 2 groups displayed a large genetic variability as compared with other groups.

We detected a significant correlation between the pairwise genetic distance and geographical distance matrices, which is indicative of isolation by distance (IBD). We also highlighted a longitudinal gradient along SWF using a multivariate technique (PCoA) based on a genetic distance matrix with SNP data from 158 landraces, which led to a significant correlation coefficient between the first PCoA axis and the longitudinal coordinates of their prospection sites.

The geographical trend observed in the diversity of SWF landraces could have been the result of diversifying selection. The occurrence of a genetical longitudinal gradient along the Pyrenees area as observed in this study has been reported for several species [46–49]. Gradients, such as altitude or longitude, play an important role in the Pyrenees, as they shape the existing proportion of suitable habitats along the mountains [47, 48]. Valbuena-Ureña *et al*. [49] emphasized that longitude is an important gradient to take into account in the Pyrenees, because the influence of the Atlantic ocean provides cooler and wetter climate westwards than in Eastern areas, which are more influenced by the Mediterranean climate.

We found differences for temperature and precipitation between the Eastern and Western parts of SWF, but regression analysis showed that, when considering geographical covariates, climatic variations did not explain genetic variability observed between SWF landraces. Since climate variation are geographically linked with longitude and latitude, we still cannot completely exclude the involvement of climatic variation in the differentiation of SWF landraces into two genetic groups; our results rather suggest that an equilibrium between restricted gene flow and genetic drift is the main driver of population differentiation between landraces in this region. This is supported by the longitudinal and latitudinal gradients that we observed on the first axis of PCA, which is determined by a large number of SNPs. However, the genetic differences observed for SWF landraces could be also explained by farmers’ practices: the management of seed exchanges in different valleys limited by hilly mountains probably contributed to differentiate SWF landraces in different districts. However, the ethnobotanical survey realized in 2012–2013 among farmers in the Pyrenean area did not reveal any major differences in farmers’ practices between the East and West parts of the Pyrenees [50]. As the differenciation signal can be seen at the genomic level, its suggests that demographic and gene flow forces were at work in SWF evolution.

To elucidate the origin and evolution of maize landraces in the South-West of France, we conducted an investigation of the relationship between SWF and putative ancestors.

### The use of genome wide analysis to infer the origin of SWF landraces

#### A predominant origin from Pyrenees-Galicia

We did not observe any clear differentiation between SWF landraces and those of the Pyrenees-Galicia group, suggesting that SWF landraces originated from a single genetic group close to the ancestors of the Pyrenees-Galicia group (*F*_ST_ = 0.03 between the Pyrenees_Galicia_2 group and each of the groups from the South-West of France) [4, 7, 8, 10].

Several studies on genetic diversity of European and American maize landraces [4, 5, 7] highlighted a Pyrenees-Galicia Flint group made up of European landraces including a small sample of landraces from the South-West of France and landraces from the North of Spain. This original landrace group was hypothesized to originate from hybridizations between landraces of Caribbean and Northern Flint groups; however less than 30 landraces from Pyrenees-Galicia were used in these previous studies. In our study, using a much larger data set of 194 maize landraces from the SWF, the origin for SWF landraces was more precisely analysed. Our PCA and Neighbor-joining analyses showed genetic proximity between landraces from the Western part of the South-West of France and maize landraces from PG_2 group, supporting genetic relationship mentioned between landraces from Pyrenees (France and Spain) and Galicia in previous studies of maize landraces named as Pyrenees-Galicia Flint group [7, 9, 10]. Based on historical documents, the introduction of maize in the South-West of France was reported by Renoux [22] as originating from Spain. In her study, Vouette [51] reported the name of maize in the Pyrenees valleys as "wheat from Spain", as given by Bonnafous (1836). Similarly, maize is first mentionned in the "Mercuriales of Verfeuil" (near Toulouse) in 1637 as "millet of Spain" [52]. The vernacular names thus only gave indications of where the maize landraces came from before their introduction to a given country. The introduction of SWF maize from Spain in the 17th century is also explained by the coasting trade practiced by fishermen from French and Spanish fishing harbours on the Atlantic coast [53]. In his work on relations between Spain and the South of France in the 17th century, Mauro [53] described the route of maize that progressed from the West part towards the East part of South-West France. This progression of maize from West to East is consistent with the greater genetic allelic richness observed in the W-SWF group than in the E-SWF group.

#### A specific influence of Northern Flint landraces in E-SWF

NF landraces played an important role in the evolution of SWF landraces and more generally in the adaptation of maize in Europe [4, 6] and in the development of CBD germplasm [54]. Our TreeMix 3-population analysis allowed us to confirm Northern Flint landraces influence on the evolution of E-SWF group landraces. For all admixture events highlighted in this analysis, E-SWF landraces displayed a mixed origin between NF landraces with either CAR, AND or ITA landrace groups. There are two possible ways to explain the presence of NF marks in the genome of SWF landraces: either directly from a secondary introduction from North America (NFA), or from an introduction from Northern Europe (NFE) as reviewed by Tenaillon and Charcosset [13]. Revilla et al [6] supported the NFA hypothesis by suggesting that NF landraces could have been introduced several times to the North of Spain from the beginning of the 17th century. Later, Brandenburg et al. [12] found that European Flint inbred lines, *i*.*e*. first-cycle inbred lines directly derived from landraces after few generations of selfing, were issued from an admixture between inbred lines belonging to European Northern Flint landraces and those derived from Southern Spain lines. In our TreeMix 3-population test, we found a predominant involvement of the NFA group in the origin of the E-SWF group. However, our PCA analysis showed a closer genetic proximity between maize landraces from the Eastern part of SWF and Northern Flint landraces located in the North of Europe (NFE), supporting the scenario involving some gene flow from European Northern Flint landraces. These observations are probably due to the genetic similarity between NFA and NFE (*F*_ST_ = 0.04), which makes it difficult to distinguish between the two sources.

#### A limited influence fromrecent introduction of CBD varieties

Our PCA analysis showed genetic proximity between 10 SWF landraces and landraces belonging to the Corn Belt Dent genetic group (CBD). CBD is a group of landraces identified in previous studies as resulting from hybridizations between Northern Flint and U.S Southern Dent materials [41, 54]. The presence of CBD genomic traces in SWF can be explained by the recent introduction of American hybrid seeds in Europe, since hybrids belong to the CBD group. Indeed, starting in1948 with the Marshall plan, the first American hybrids were tested in France [22]. Then, from 1957 onwards, the first double hybrids with at least one American parental line were developed in France by INRA. Farmers‘ adoption of hybrids was fairly rapid [55]. These 10 landraces, showing a genetic proximity with CBD group on the PCA analysis, shaped a specific group with admixture analysis at k = 3. Introgression of CBD germplasm into local landraces has also been observed in Central Italy with very variable effects [56].

#### Other relationships with American landraces

In our TreeMix 3-population test, we found that the E-SWF group originated from crosses between NF and Andean landraces. Mir *et al*. [11] showed that SWF maize landraces had a Northern South America origin, but our study does not allow us to really assess this path of introduction, as only four maize landraces from such origin were used in this study: VEN405 from CAR, VEN736 from ITA, ANTI392 and PANA168 both from AND groups. Our PCA and Neighbor-joining analyses showed little genetic similarity between W-SWF landraces and those from Northern South-America. It is possible that our study did not capture very well genetic relationships between SWF landraces and these expected Northern South-American parental landraces identified by Mir *et al*. [11] because of a limited sample of South America landraces. In consequence, we had some difficulty to evidence relationships between SWF and Northern South America landraces. Note that American landraces introduced in Europe evolved in America during about 500 years, which limits the ability of TreeMix 3-population test to detect hybridization events between SWF and American landraces.

Our collection of SWF landraces presented the lowest genetic differentiation with landraces from Chile (except for CHZMO8050) compared to the remaining American landraces. All these Chilean landraces have been shown to originate from different genetic groups [10], with a probable replacement of traditional landraces with relatively recently introduced Northern US materials [3]. Genetic relatedness between Chilean and SWF landraces may be due to the introduction of SWF genepools into Chile or vice-versa. Interestingly, the Spanish (especially Basques and Andalusians) and also other Europeans such as French people (mainly coming from SWF) immigrated mainly to Chile in the second half of the 19th century, although Basque presence in Chile began in the conquistador period. Also exchanges between the Basque country and the South West of France have been taking place for a few centuries (https://en.wikipedia.org/wiki/Immigration_to_Chile, march 2020).

### The different scenarios for SWF landrace origin and evolution

More generally, we thought that the South-West of France had been submitted to several introductions of maize with various genetic origins, as already reported for maize landraces in Spain [6] and in Portugal [11, 57]. Evidence for subsequent admixture between the genetic groups involved in these introductions was observed in this study with TreeMix 3-populations tests, PCA analyses and genetic distance analyses. This is particularly true for E-SWF landraces that showed hybridization between landraces from NF and either ITA, or CAR or AND groups in TreeMix 3-populations tests. E-SWF landraces were more scattered than W-SWF landraces, which were more homogenous. The hybridization of E-SWF group built by ITA and NFA groups could be explained by the presence of Italian agricultural workers in the 19^th^ and 20^th^ centuries in SWF [58].

All the results above allowed us to describe at least two scenarios of origin that take into account the influence of Northern Flint landraces from the North of Europe predominently in the Eastern part and the genetic proximity of W-SWF landraces with Tropical ones. For the first scenario, we postulated that, after the introduction of hybridized maize landraces from Spain (Caribbean x Northern Flint landraces, known as Pyrenees-Galicia group), the NFE landraces spread from the North to the Eastern part of SWF where they hybridized together.

In the second scenario, we hypothesized that the first maize landraces introduced to the South-West of France displayed predominantly Northern Flint ancestries as do Galician landraces [11]. Thus, these landraces spread from the Western to the Eastern parts of South-West France. Thereafter, Tropical landraces (from Mexican, Caribbean and Andean genetic groups) would have been introduced secondarily into Western altlantic coast spanning possibily Cantabrian and Basque coasts and hybridized with local landraces forming a new genetic entity which migrated from W-SWF to E-SWF. The gene flows were restricted as supported by IBD indicator, which slowed down the West-East introgression, explaining the current differenciation pattern between W-SWF and E-SWF. Presence of genetic marks from Tropical landraces also resulted from seed exchanges between Mediterranean trademen from Italy, France, Spain and Portugal at the end of the sixteenth century [11, 12]. Revilla Temiño *et al*. [5], Patto *et al*. [57], Brandenburg *et al*. [12] and Mir *et al*. [11] found that the South of Europe has experienced maize landraces introductions from both the Caribbean islands and South America.

The contribution of maize landraces from various genetic groups found in this study makes it difficult to determine which one of the two plausible scenarios mentioned above best explains the origin of SWF landraces. Additional historical data on maize introduction to Northern Spain and SWF would certainly help to shed more like light on this aspect.

## Conclusion

Assessing the population genetic structure and diversity of a collection of crop accessions is the best way to develop an efficient management strategy and to improve breeding programs. Here we used DNA bulk analyses with a 50K SNP array to investigate the diversity and population structure for a panel of 194 maize landraces collected 30 to 60 years ago in the South-West of France. In this study, we assumed that the maize landraces from this area mostly originated from hybridizations between landraces from the Caribbean and Northern Flint groups. Introduced around 1626 through Atlantic harbours such as Bayonne, these SWF landraces have probably been posteriously submitted to gene flows from landraces belonging to various countries of America and Europe, as expected by Revilla Temiño *et al*. [5] and Patto *et al*. [57] for Spanish and Portuegese landraces respectively. A single mixture of NF and CAR landraces is not sufficient to explain the genetic origin of SWF maize.

The new results obtained for SWF in our study are encouraging to expand the approach to other regions of the world, to get a better understanding of diversity and history of local varieties.

## Supporting information

S1 Fig Plot of DeltaK of the log likelihood from the admixture analysis on SWF landraces SNP data. Group numbers varied from K = 1 to K = 13.
(TIF)

S2 Fig Bar-plot for K = 2 to K = 5 for admixture analysis with the 194 SWF maize landraces. At K = 2, we differentiated W-SWF (in cyan) and E-SWF (in blue) genetic groups. At K = 3, 15 landraces (in dark-grey) previously assigned to E-SWF group at K = 2 constituted a group distinguished from E-SWF and W-SWF groups. At K = 4, we observed a fourth group consisting of about 10 landraces located principally in the “Lot” and in “Lot et Garonne” districts. At K = 5, landraces from Gironde (in turquoise) differ from W-SWF groups; Landraces from the “Lot” and “Lot et Garonne” districts were integrated again in E-SWF group and we observed differentiation between landraces from the third group at K = 3.
(TIF)

S3 Fig Heat-map of Rogers ‘genetic distance values between the 194 landraces from SWF and the 82 landraces from America. American landraces were sorted according to the latitude of their collection sites and colored as per their genetic groups previously identified by Camus-Kulandaivelu et al. [10]. We also sorted SWF landraces using their ancestries values on W-SWF group obtained with admixture analysis at K = 2; thus SWF landrace numbers from 0 to 65 represent the E-SWF landrace group and SWF landrace numbers from 66 to 194 represent the W-SWF landrace group. Corn Belt Dent in red, Caribbean in green, Northern Flint in yellow, Mexican in brown, Italian Flint in orange, Andean in magenta and the 9 landraces from South America studied by Mir et al (2017) in black.
(TIF)

S1 Table Combination of passport data (column 1 to 5), genetic diversity analysis (column 6 and 7) and admixture analysis at K = 2 (column 8 to 10) performing on SNP data of the 194 maize landraces from the South-West of France. W-SWF = "West South-West France»; E-SWF = " East South-West France".
(XLSX)

S2 Table Passport information and PCA analysis result on SNP data of 342 maize landraces. (XLSX)

S3 Table Climatic variations between E-SWF and W-SWF groups from May to October. This table contained means of temperature and precipitation for W-SWF and E-SWF groups from May to October. We shaded in bold months for which differences for mean temperatures and precipitations between the two groups were significant (*p-value*<5%).
(XLSX)

S4 Table TreeMix 3-population test results. This table represents a summary of TreeMix 3-population test results performed using all combinations of three groups shaped by each of the two groups from SWF (E-SWF and W-SWF) considered as focal group, and two other groups (Reference groups 1 and 2) considered as the two potential ancestors of the focal group. MEX = Mexican, AND = Andean, CAR = Caribbean, NFA = Northern Flint material from America, NFE = Northern Flint material from Europe, CBD = Corn belt dent, ITA = Italian, E-SWF = East South-West France, W-SWF = West South-West France.
(XLSX)
