# Supplementary figures and images for: Genetic diversity of maize landraces from the South-West of France
Source: PLoS One. 2021 Feb 1;16(2):e0238334. doi: 10.1371/journal.pone.0238334 (PMC7850504; doi:10.1371/journal.pone.0238334)

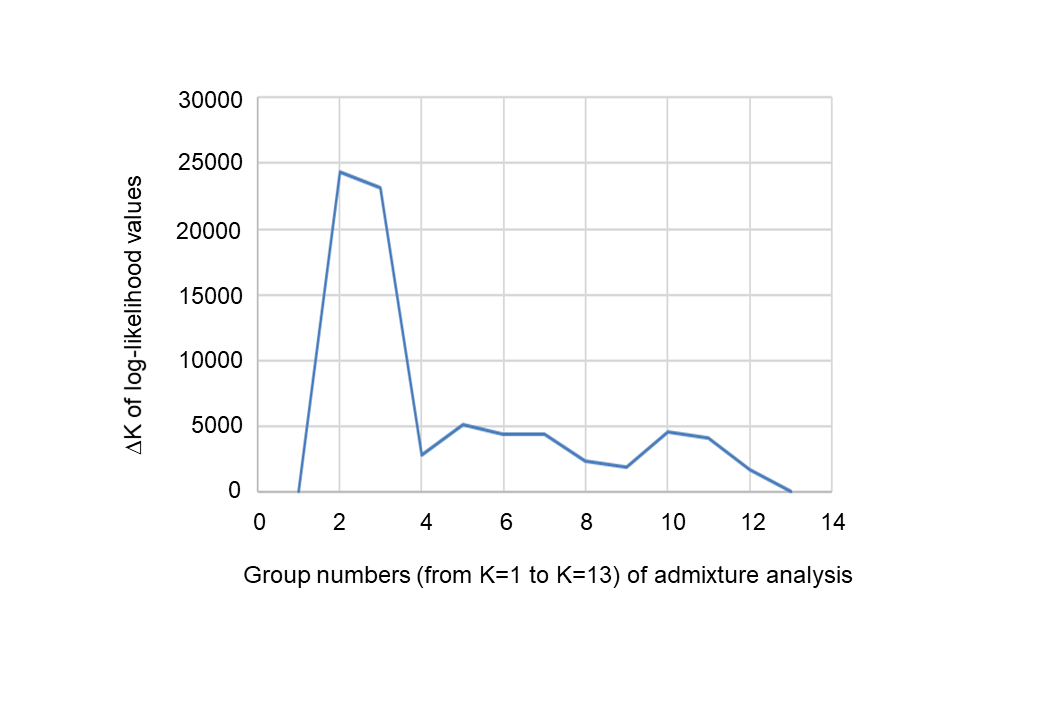

Supplement: S1 Fig — Group numbers varied from K = 1 to K = 13. (TIF) [file pone.0238334.s001.tif]

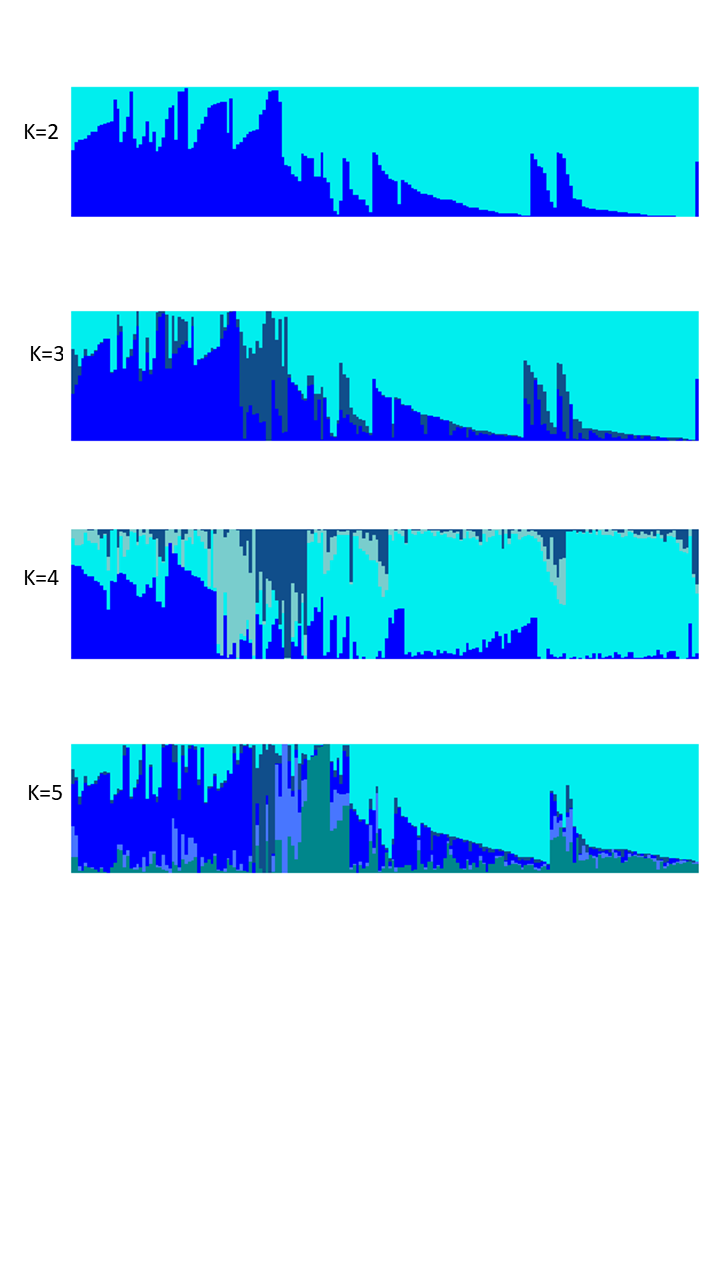

Supplement: S2 Fig — At K = 2, we differentiated W-SWF (in cyan) and E-SWF (in blue) genetic groups. At K = 3, 15 landraces (in dark-grey) previously assigned to E-SWF group at K = 2 constituted a group distinguished from E-SWF and W-SWF groups. At K = 4, we observed a fourth group consisting of about 10 landraces located principally in the “Lot” and in “Lot et Garonne” districts. At K = 5, landraces from Gironde (in turquoise) differ from W-SWF groups; Landraces from the “Lot” and “Lot et Garonne” districts were integrated again in E-SWF group and we observed differentiation between landraces from the third group at K = 3. (TIF) [file pone.0238334.s002.tif]

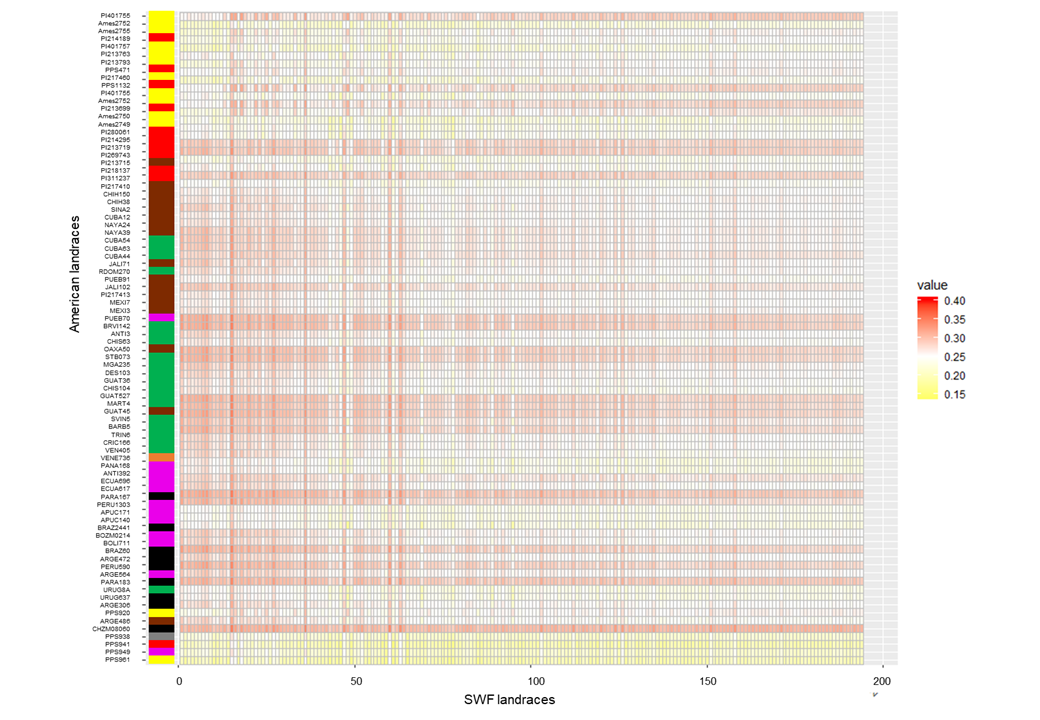

Supplement: S3 Fig — American landraces were sorted according to the latitude of their collection sites and colored as per their genetic groups previously identified by Camus-Kulandaivelu et al. [10]. We also sorted SWF landraces using their ancestries values on W-SWF group obtained with admixture analysis at K = 2; thus SWF landrace numbers from 0 to 65 represent the E-SWF landrace group and SWF landrace numbers from 66 to 194 represent the W-SWF landrace group. Corn Belt Dent in red, Caribbean in green, Northern Flint in yellow, Mexican in brown, Italian Flint in orange, Andean in magenta and the 9 landraces from South America studied by Mir et al (2017) in black. (TIF) [file pone.0238334.s003.tif]
